# 640. evaluation of Carbapenemas-producing enterobacterales (CPE) clearance protocol in A secondary hospital in Israel

**DOI:** 10.1093/ofid/ofaf695.204

**Published:** 2026-01-11

**Authors:** Pnina Shitrit, Anna shoyket, yael pri-Paz Basson

**Affiliations:** Meir Medical Center, Kfar-Saba, Tel Aviv, Israel; Meir medical center, kfar saba, Tel Aviv, Israel; Meir Medical Center, Kfar-Saba, Tel Aviv, Israel

## Abstract

**Background:**

Carbapenemase-producing Enterobacterales (CPE) are difficult to treat, and both colonization and infection rates are increasing globally. In Israel, CPE carriers are isolated under strict contact precautions, which may lead to emotional distress and an increased risk of medical errors and non-infectious adverse events (1).

Previous studies have shown that a significant proportion of patients experience spontaneous decolonization of CPE (2), which allows discontinuation of isolation. Our clearance protocol, based on national guidelines, includes patients who are at least three month from a positive test and were not hospitalized during this period., clearance required three negative tests, including one polymerase chain reaction (PCR) (3, 4). We aimed to evaluate the effectiveness of national clearance protocol implementation in our hospital and to find risk factors for clearance failure or re-colonization

**Figure:**
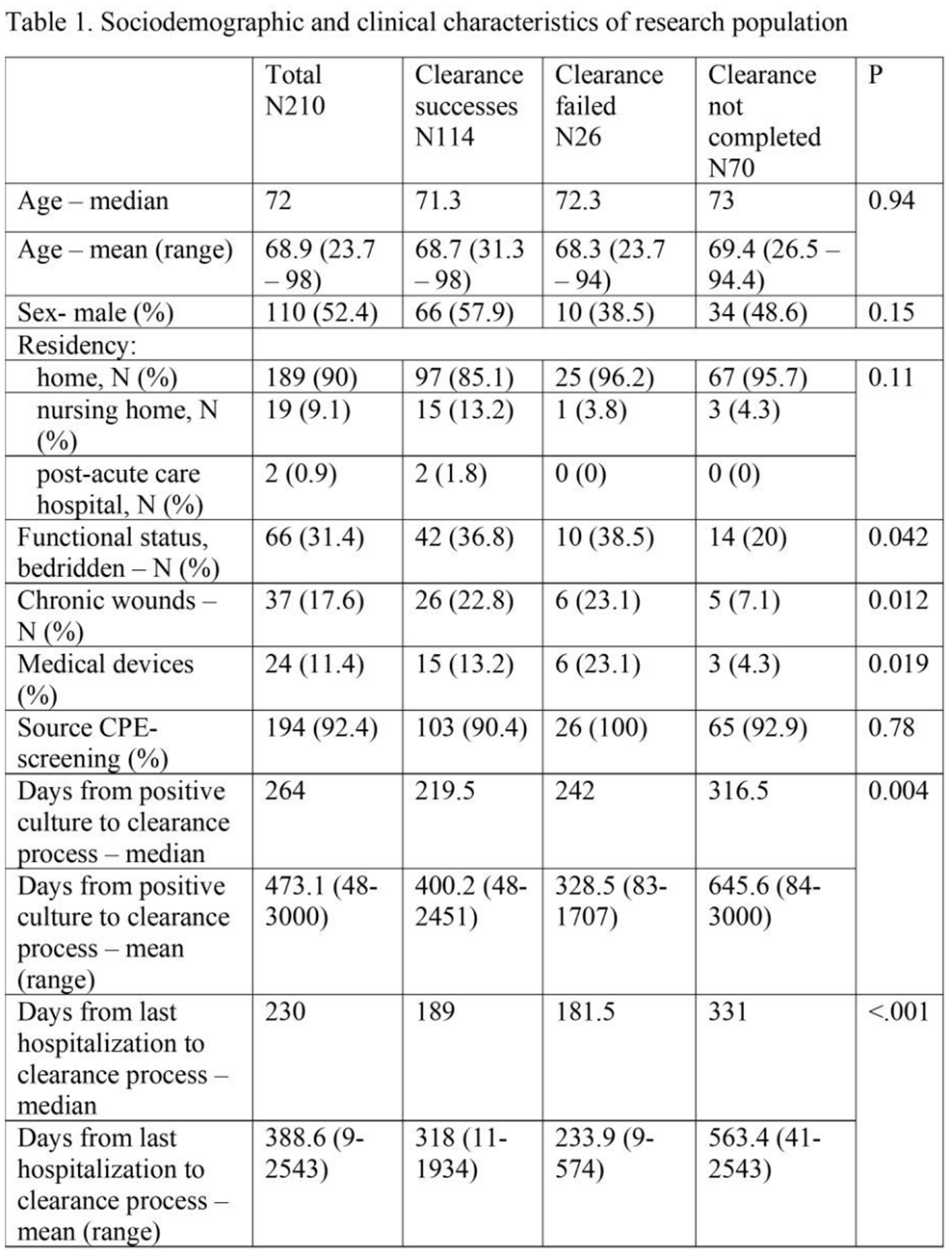

**Figure:**
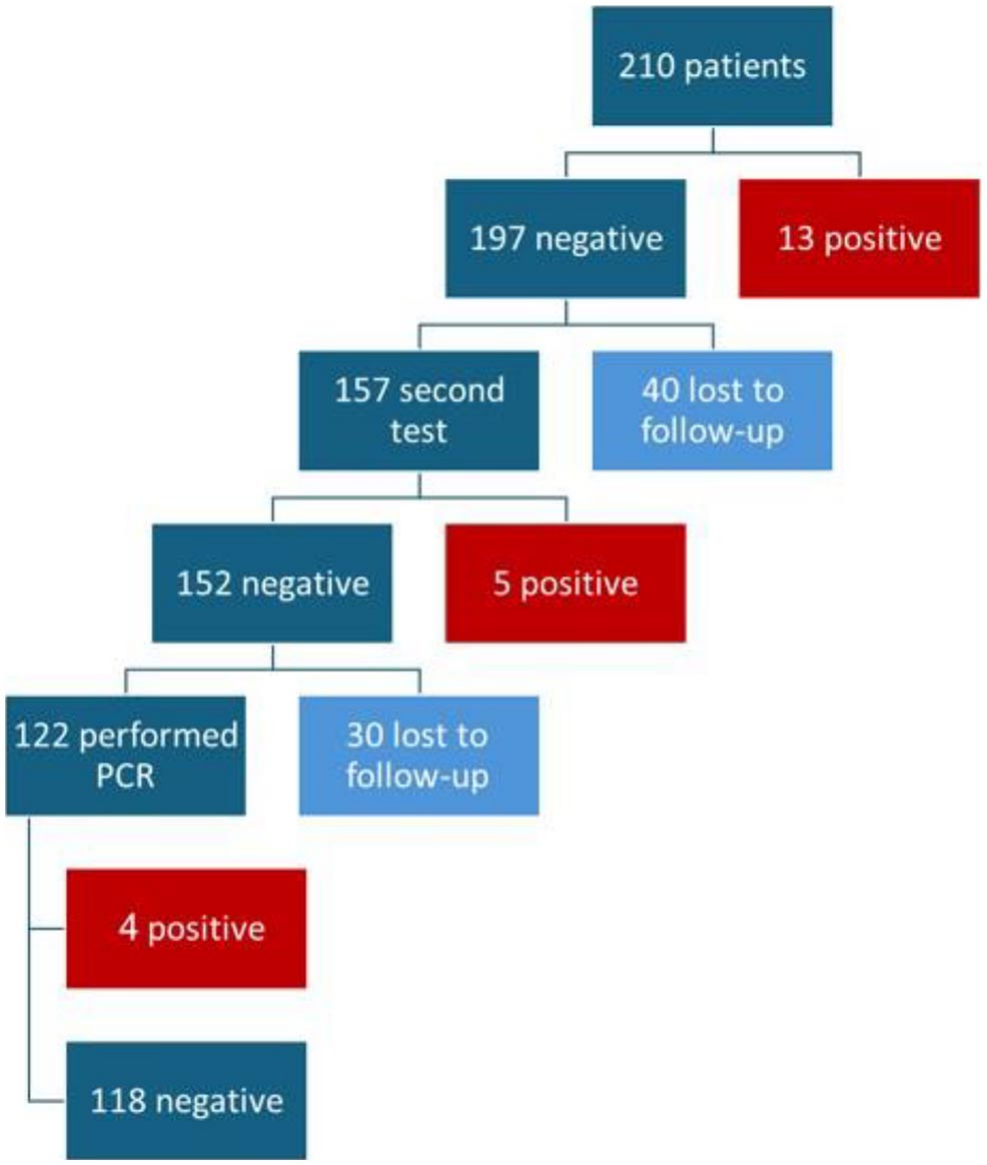

**Methods:**

Patients with CPE-positive rectal screening or clinical culture who met clearance criteria were included. Demographic data, functional status, place of residency, and underlying conditions were collected from medical records. Microbiological data included date of first and last positive CPE screening, organism, and the resistance mechanism.

**Figure:**
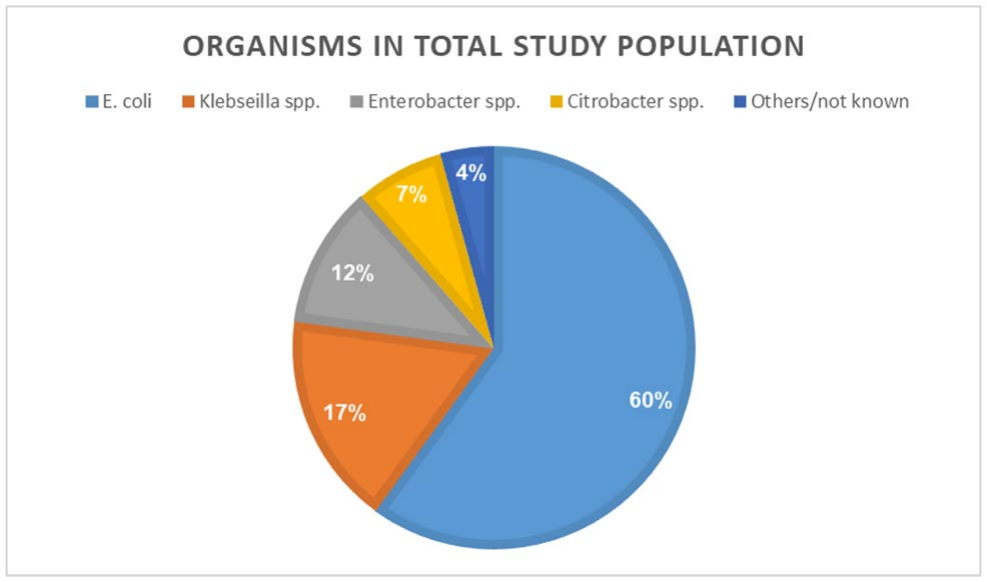

**Figure:**
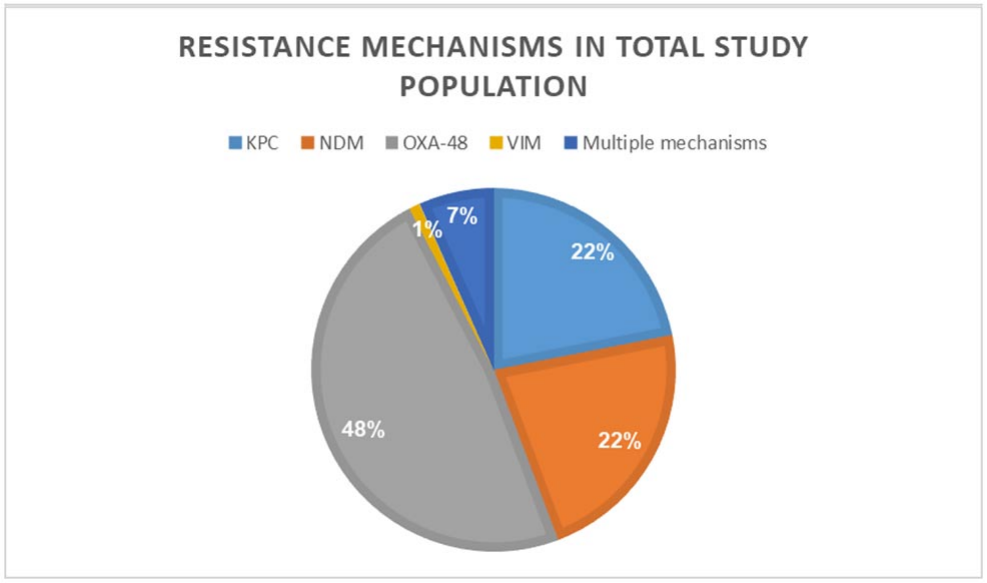

**Results:**

From 2014 to 2024, 210 CPE patients who met clearance criteria were included. 110(52.4%) were males and average age was 68.9 years (range 23.7-98). 189 (90%) lived at home and 144(68.6%) were functionally independent. 92.4 % of the CPE cases were identified through screening cultures. Of the 140 patients (66.7%) who completed the protocol, 114(81.4%) were cleared, 22(15.7%) had positive culture with the same mechanism and 4(2.8%) were recolonized with different mechanisms. Compared to patients who were cleared or failed the protocol, those who did not complete the protocol had fewer wounds (7.1% vs. 23.1%/22.8%, p=0.012), fewer devices (4.3% vs. 23.1%/13.2%, p=0.019), were less often bedridden (20% vs. 38.5%/36.8%, p=0.042), and had a longer interval from acquisition to protocol initiation.

**Conclusion:**

CPE clearance protocol, based on national Israeli guidelines is highly effective. Patients with lower medical dependency were less likely to complete the process

**Disclosures:**

All Authors: No reported disclosures

